# CD73/NT5E-mediated ubiquitination of AURKA regulates alcohol-related liver fibrosis via modulating hepatic stellate cell senescence

**DOI:** 10.7150/ijbs.80461

**Published:** 2023-01-16

**Authors:** Zhenni Liu, Baoming Wu, Xueqi Liu, Xue Wu, Jiyu Du, Guoqing Xia, Junnan Cai, Hong Zhu, Xiaodong Sheng, Mengda Zhang, Junrui Xu, Tao Xu, Xiongwen Lv

**Affiliations:** 1 Inflammation and Immune Mediated Diseases Laboratory of Anhui Province, Anhui Institute of Innovative Drugs, Hefei, China.; 2 School of Pharmacy, Anhui Medical University, Hefei, China.; 3 Institute for Liver Diseases of Anhui Medical University, Hefei, China.; 4 General Thoracic Surgery, the First Affiliated Hospital of Anhui Medical University, Hefei, China.

**Keywords:** Alcohol-related liver fibrosis (ALF), CD39-CD73-Adenosine axis, Aurora kinase A (AURKA), p53 signaling pathway

## Abstract

Alcohol-related liver disease (ALD) is the most common chronic liver disease worldwide; however, no effective treatment to prevent the progression of alcohol-related liver fibrosis (ALF) is available. CD73/NT5E, a nucleotidase, controls cellular homeostasis by combining extracellular purinergic signaling with intracellular kinase activity and gene transcription and is associated with cell proliferation, differentiation, and death. In this study, we demonstrated that CD73/NT5E had a more significant regulatory effect on the activation, proliferation, and apoptosis of HSCs compared with that of CD39/ENTPD1. We examined the expression of CD73/NT5E in the normal and fibrotic human livers. The absence of CD73/NT5E was protective in mouse models of ALF. In addition, Kyoto Encyclopedia of Genes and Genomes (KEGG) pathway analyses showed that CD73/NT5E overexpression was related to the p53 signaling pathway, which regulates cell senescence. Proteins interacting with p53 were predicted using the STRING database. The overlap between proteomic analysis and STRING databases was for Aurora kinase A (AURKA), a cell cycle-regulated kinase. Coimmunoprecipitation (co-IP) assay and molecular docking confirmed that CD73/NT5E directly interacted with AURKA. We found that overexpression of CD73/NT5E inhibited AURKA ubiquitination, whereas p53 signaling was downregulated. Mechanistically, CD73/NT5E regulated ALF and the activation and senescence of stellate cells by binding to AURKA. These findings indicate that CD73/NT5E is a potential therapeutic target for ALF.

## Introduction

Alcohol-related liver disease (ALD) is the most prevalent type of chronic liver disease and poses a significant disease burden worldwide 1. Alcohol consumption has become a global phenomenon 2. Owing to the improvement in the quality of life and the explosive growth of the economy in China, the consumption of alcohol has increased significantly, with an associated increase in ALD incidence 3, 4. Excessive alcohol consumption is a major cause of chronic liver disease, leading to simple steatosis, steatohepatitis, fibrosis, cirrhosis, and hepatocellular carcinoma (HCC). Despite extensive research on ALD in recent years, no effective targeted therapy has been identified 5, 6.

ALD pathogenesis includes hepatic steatosis, oxidative stress, acetaldehyde-mediated toxicity, and cytokine and chemokine-induced inflammation 7. The current academic view holds that fibrotic repair is a key link in the reversible recovery of ALD 8, 9. Alcohol-related liver fibrosis (ALF) is a scarring reaction caused by long-term alcohol consumption and is characterized by the deposition of a large amount of extracellular matrix (ECM) in the liver. Hepatic stellate cells (HSC) play a central role in the pathogenesis of liver fibrosis, transdifferentiating in chronic liver disease from “quiescent” HSC to proliferative, migratory, and fibrogenic myofibroblasts that exhibit profibrotic transcriptional and secretory properties (so-called “cellular activation”), and secretes a large number of ECM proteins, tissue inhibitors of metalloproteinases, and matrix metalloproteinases (MMPs) that trigger structural remodeling of the liver 10, 11. Therefore, HSC depletion is critical for fibrosis resolution.

Purine signaling is mainly composed of three parts: extracellular nucleotides, ectonucleotidases (such as CD39 and CD73), and specific receptors that act on nucleotides and their derivatives 12. Among them, CD39 (ectonucleoside triphosphate diphosphohydrolase-1, ENTPD1) and CD73 (ecto-5′-nucleotidase, NT5E) are key regulatory molecules in the purine signaling pathway. ATP is rapidly hydrolyzed by CD39 and CD73 to adenosine when released from stressed or damaged cells into the extracellular space 13. As a bridge to maintaining the balance of purinergic signaling, the role of the CD39-CD73-adenosine signaling pathway in fibrotic diseases has attracted the attention of many researchers. Fernández et al. reported that deletion of the enzymes involved in adenosine production (CD73, CD39, or both) prevents skin fibrosis in murine models 14. Preventing the increase in extracellular adenosine levels by inhibiting the function of CD39 and/or CD73 may attenuate the fibrogenic effects of adenosine. However, some studies have provided evidence to the contrary. Peng et al. reported that knockout of CD39 in mice with sclerosing cholangitis promoted biliary tract injury and fibrosis 15.

Our previous experiments showed that the pharmacological blocking of CD73 or CD39 expression could inhibit the activation and proliferation of HSCs through the Wnt/β-catenin or TGF-β/Smad3 signaling pathways. In this study, we compared the roles of CD39 and CD73 in HSC activation and proliferation. We further tested the role of CD73 in alcohol-related liver fibrosis *in vivo* in CD73 knockout (KO) mice and *in vitro* in CD73-overexpressed and CD73-silenced HSCs/LX-2 cells. The downstream target of CD73 was also identified.

## Materials and methods

### Reagents and materials

Antibodies included anti-β-actin (Santa Cruz Biotechnology, sc-47778), anti-α-SMA (Bioss, bs-10196R and Bioss, bsm-33187M), anti-COL1a1 (Bioss, bs-10423R), anti-CD39 (proteintech, 14211-1-AP), anti-CD73 (Invitrogen, PA5-81614 and proteintech, 12231-1-AP), anti-c-Myc (Abcam, ab32072), anti-Cyclin D1 (Abcam, ab40754), anti-Bax (Abcam, ab32503), anti-Bcl-2 (Abcam, ab182858), anti-cleaved caspase 3 (Abcam, ab184787), anti-AURKA (Wanleibio, WL03733 and Abcam, ab52973), anti-p-AURKA (Abmart, TA3011), anti-p53 (Bioss, bs-8687R), anti-p21 (Abcam, ab109199) and anti-p16 (Wanleibio, WL01418). HRP-conjugated anti-rabbit secondary antibodies were obtained from ZSGB-BIO. MLN8237 was purchased from ApexBio Technology (A4110). Cycloheximide (CHX), MG132, chloroquine (CQ) and N-[N-(N-Acetyl-L-leucyl)L-leucyl]-L-norleucine (ALLN) were purchased from Selleck. ALT assay kit and AST assay kit were obtained from Mindray. The Senescence beta-Galactosidase Staining Kit was obtained from Beyotime (C0602).

### Human liver tissue samples

The non-tumor portion of the liver were obtained from a patient who had undergone partial hepatectomy and had a history of drinking alcohol. The degree of fibrosis was classified as normal liver and mild to moderate fibrosis according to the Liver Cancer Study Group of Japan 16. This study was approved by Biomedical Ethics Committee of Anhui Medical University. And all protocols adhered to the principles outlined in the Declaration of Helsinki.

### Animal studies

**Figure 1:**
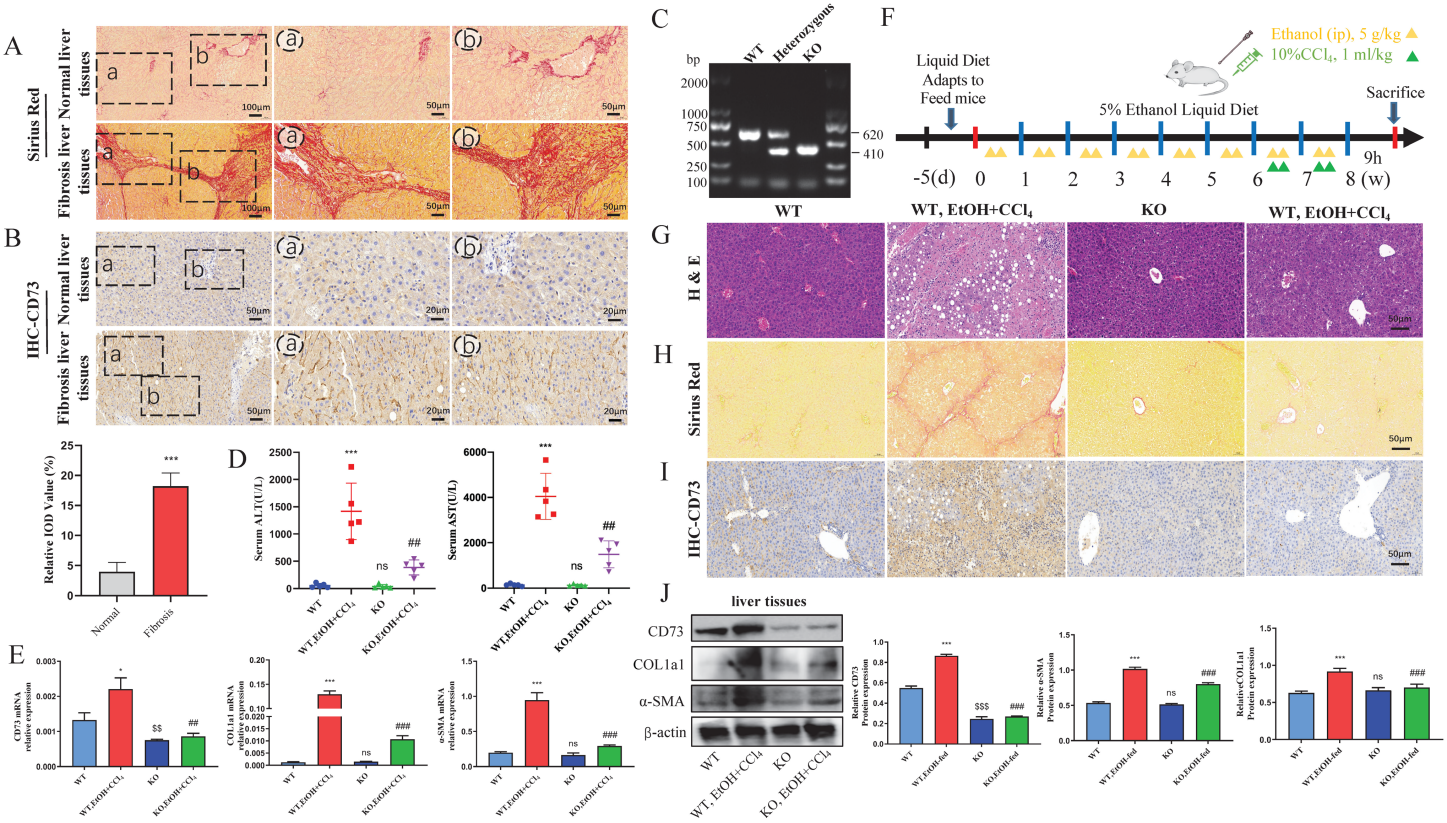
** KO of CD73 protected against EtOH+ CCl_4_-induced liver injury and fibrosis in mice. (A)** Representative pictures of Sirius Red staining of human liver tissue sections were shown (50 μm and 100 μm). **(B)** Representative pictures of CD73 immune staining of human liver tissue sections were shown (20 μm). **(C)** CD73 deficiency was confirmed by assessing genomic DNA. **(D)** Serum ALT and AST levels were measured. The mRNA **(E)** and protein** (J)** levels of CD73, α-SMA and COL1a1 in liver tissues were measured by Western blot and RT-qPCR. **(F)** Establishment of an alcohol-related liver fibrosis model. Representative pictures of H&E **(G)** and Sirius Red staining **(H)** of liver tissue sections were shown (50 μm). **(I)** Representative pictures of CD73 immune staining were shown (50 μm).^ *^P < 0.05, ^***^P < 0.001 vs. the WT group. ^$$^*P* < 0.01, ^$$$^*P* < 0.001 vs. the WT group. ^##^*P* < 0.01, ^###^*P* < 0.001 vs. the WT, EtOH-fed+CCl_4_ group.

Male C57BL/6 mice (aged 8-12 weeks) were obtained from the Experimental Animal Center, Anhui Medical University. All experimental procedures in this study were approved by the Animal Experimentation Ethics Committee from Anhui Medical University, Anhui, China. The mice in model group were fed a liquid diet containing 5% ethanol for 8 weeks, and were gavaged with a high-concentration alcohol (5 g/kg) twice a week. In the last two weeks, mice received intraperitoneal injections of CCl_4_ (10 % in olive oil, 1 ml/kg). The liver tissues and blood from these mice were collected after the last alcohol gavage for further analysis and primary hepatic stellate cells (HSCs) were isolated (Fig. 1F). CD73 knockout mice were obtained from the Cyagen Biosciences Inc. Mice were divided into four groups: (i) WT (ii) WT, EtOH-fed+CCl_4_ (iii) KO (iv) KO, EtOH-fed+CCl_4_. All mice were genotyped by PCR before and after experiments.

### Primary HSCs Isolation

Hepatic stellate cells (HSCs) were isolated by *in situ* collagenase perfusion of the liver. Briefly, mice were anesthetized, a 24-G catheter was put through mouse the portal vein and the inferior vena cava were cut. Then the liver was perfused with PB until the liver was free of blood. Replace the PB with the freshly prepared enzyme solution, and clamp the inferior vena cava with hemostatic forceps. After the liver was completely digested, removed it and placed it into a glass dish containing 1% BSA. The cells were liberated by tearing and shaking of the liver with forceps. Cells suspension was centrifuged at 4 °C, 50 g, 2 min. The supernatant was collected and centrifuged at 4 °C, 760 g for 10 min. The supernatant was discarded, and the pellets were resuspended in 4 ml DMEM, and the previous step was repeated. The pellet was resuspended with 2 ml DMEM for use. The cell suspension was subsequently mixed with Nycodenz (Sigma, GER) solution to give a final density between 1.04 to 1.06 g/ml by adding DMEM. Nycodenz mixture was covered with 1ml Hank's fluid (Gibco, United States) and centrifuged at 20 °C, 1350 g for 18 min. After the centrifugation, the cells at the interface were transferred to a new 15 ml tube, then an appropriate amount of DMEM was added, centrifuged at 1350 g for 5 min, the pellet was resuspended in 3 ml DMEM, and inoculated into culture bottles.

### Liver histology and immunohistochemistry

Paraffin-embedded mouse liver sections were prepared by a routine procedure including fixation, dehydration, waxing, and embedding. The fixed sections were 4 μm thick and then stained with hematoxylin and eosin (H&E) staining, Masson staining and Sirius Red staining. Immunohistochemical staining was performed according to the manufacturer's instructions. The antibodies specific for CD73 (proteintech, 12231-1-AP), CD39 (proteintech, 14211-1-AP), and AURKA (Wanleibio, WL03733), α-SMA (Bioss, bs-10196R) were incubated overnight at 4 °C, and the secondary antibodies were incubated for 1 hour at room temperature. Then staining was observed with 3,3-diaminobenzidine tetrahydrochloride (DAB) staining. Slides were visualized using a microscope (Olympus IX83, Japan).

### Immunofluorescent staining

The sections were permeated with 1 % Triton X-100 for 15 min. In total, 5% BSA was used for tissue blocking. The frozen liver tissue sections were incubated with rabbit polyclonal primary antibodies for CD73 (proteintech, 12231-1-AP)/ CD39 (proteintech, 14211-1-AP) mouse monoclonal primary antibodies for α-SMA (Bioss, bsm-33187M)/ F4/80 (Santa Cruz Biotechnology, SC-377009)/ CK19 (Santa Cruz Biotechnology, SC-376126) overnight at 4 °C, the cell sections were incubated with rabbit polyclonal primary antibodies for AURKA (Wanleibio, WL03733) mouse monoclonal primary antibodies for CD73 (Invitrogen, PA5-81614), and then incubated with a secondary antibodies for 2 h. The stained sections were visualized and captured using a microscope (Olympus IX83, Japan).

### ALT/AST/Adenosine activity

The contents of ALT and AST in serum were measured by automatic biochemical analyzer. Take 10 μl of serum and put it into a 1.5 ml EP tube with 90 μl of PBS. The EP tube, R1 and R2 reagents were put into an automatic biochemical analyzer to detect the content of ALT and AST. Serum adenosine content was detected according to the Adenosine Assay Kit (Abcam, ab211094).

### Western blot

The protein lysates from liver tissues and cultured cells were prepared following standard protocols, and Western blot analysis was performed as described previously 17. Primary antibodies used in this study include β-actin, α-SMA, COL1a1, CD39, CD73, c-Myc, Cyclin D1, Bax, Bcl-2, cleaved caspase 3, AURKA, p-AURKA, p53, p21 and p16. Signals were detected using a chemiluminescent (ECL) system (Bio-Rad, USA) and the results were analyzed using ImageJ software (National Institutes of Health, USA).

### Total RNA isolation and quantitative real-time PCR

Total RNA from liver tissues and cultured cells was extracted using TRIzol (Invitrogen, United States) following the manufacturer's instructions. RNA was quantified by a Nanodrop 2000 (Thermo Scientific, USA). The mRNA levels of GAPDH, α-SMA, COL1a1, CD39, CD73 and AURKA were determined by RT-qPCR. The primer sequences were listed in Table 1.

### Cell culture and treatment

Rat HSC-T6 cells line was obtained from the Type Culture Collection of the Chinese Academy of Sciences (Shanghai, China). The human HSC line (LX‐2) was obtained from Xiang Ya Central Experiment Laboratory (China). HSC-T6 cells and LX‐2 cells were cultured in DMEM (HyClone, USA) supplemented with 10% fetal bovine serum (FBS, Biological Industries, Israel). Cells were incubated at 37 °C with 5% CO_2_. HSC-T6 cells were activated by 200 μM acetaldehyde. LX‐2 cells were activated by 600 μM acetaldehyde.

### Flow cytometry

To detect apoptosis of HSC-T6 cells, the Annexin-V-FITC/PI Apoptosis Detection Kit (Best bio, China) was used according to the manufacturer's standard protocol. Cells were transferred to a flow tube and detected by Flow cytometry (Beckman Coulter, USA).

### CCK-8 Analysis

The HSC-T6 cells were collected at the exponential phase and seeded into a 96-well plate (the edge wells were filled with sterile PBS). After attachment, HSC-T6 cells were treated with different concentrations of MLN8237 (0, 1.25, 2.5, 5, 10, 25, 50, 100, 200 nM) and CHX (0, 10, 25, 50, 100, 200, 400, 600 μM) for 48 h. Then, 10 μl CCK-8 (Bioss, China) was added for 1 h. The value of absorbance (A) was examined at the wavelength of 490 nm.

### Proteomic analysis

Briefly, HSC-T6 cells transfected with pEX3-NC-CD73 and pEX3-CD73 plasmid were collected and lysed with protein lysate according to the manufacturer's instructions. LC-MS/MS data was collected using the latest generation Orbitrap mass spectrometer (Q-Exacitve HF) after protein Trypsin digestion. The data were analyzed using the Proteome Discoverer software platform. The uniprot-taxonomy 10114 (rattus+ norvegicus) fasta database was used.

### Cell transfection

To down-regulate or up-regulate the expression of CD39, CD73 and p53, CD39-siRNA/pEX-3-CD39, CD73-siRNA/pEX-3-CD73, p53-siRNA/pEX-3-p53 and corresponding control RNA were used (purchased from Hanbio and Gene Pharma). The siRNA sequences were listed in Table 2. Briefly, siRNA or plasmid was transferred into cells cultured in Opti-MEM using LipoFiterTM3 after 24 h of cell culture. After 6 h, cells were cultured in DMEM containing 10% BI and treated with acetaldehyde for 48 h and consequently harvested for analysis with Western blotting, RT-qPCR and other experiments.

### Co-immunoprecipitation assay

Cells were lysed in an ice bath with lysis buffer (Beyotime Biotechnology, China) for 30 min, and centrifuged at 3000 rpm for 15 min at 4 °C to obtain the supernatant. Cell lysates were incubated with the anti-CD73 antibodies for 2 h. Then protein A/G agarose beads (Bioworld, U.S.A) were added and the mixtures allowed incubating at 4 °C with gentle rocking overnight. Beads were washed with an appropriate amount of lysis buffer and eluted. The immune complexes were then subjected to Western blot for determination of AURKA (Wanleibio, WL03733) protein expression.

### HSCs senescence analysis

The senescence-associated β-galactosidase (SA-β-Gal) staining kit (Beyotime, Shanghai, China) was used to detect cellular senescence. Briefly, treated cells were washed with PBS, then 1 ml of staining fixative was added and fixed for 15 minutes. After that 1 ml of staining working solution was added and cells were incubated overnight at 37 °C, then observed cells under ordinary light microscope.

### Molecular docking analysis

Based on the crystal structure of CD73 and AURKA, the target proteins, CD73 and AURKA were docked using ZDOCK to find the phosphorylation site of AURKA bound to CD73. The crystal structure of CD73 and AURKA were obtained from the Protein Data Bank (PDB). The docking studies were performed using AUTODOCK 4.1 software. All docking calculations were done using a Lamarckian genetic algorithm.

### Statistical analysis

All statistical analyses were performed using GraphPad Prism 7. Results are expressed as mean ± SD from multiple experiments. Student's t-test or one-way analysis of variance (LSD) were used for statistical significance test. The differences were considered statistically significant at *p* < 0.05.

## Results

### Upregulation of CD39-CD73-adenosine axis and fibrotic indicators in EtOH+CCl_4_ feeding mice and acetaldehyde-induced HSC-T6 cells

To confirm the expression of the CD39-CD73-adenosine axis in ALF, we established an *in vivo* model. As shown in Supplementary Fig. 1A, the livers of mice in the EtOH-fed+CCl_4_ group presented with fibrotic lesions and evident hepatomegaly. In serum of EtOH-fed+CCl_4_ mice, ALT and AST levels were significantly higher than in the controls (Supplementary Fig. 1B). H&E staining showed that the livers of EtOH-fed+CCl_4_ mice exhibited excessive inflammatory cell infiltration and hepatocyte necrosis (Supplementary Fig. 1C). The degree of fibrosis in mouse livers from different groups was evaluated using Masson staining and IHC-α-SMA. As shown in Supplementary Fig. 1D and E, EtOH +CCl_4_ enhanced collagen fiber deposition and α-SMA accumulation. These results indicated that the model was successfully established. To evaluate the liver-resident cells that expressed CD73/CD39, we performed double immunofluorescence staining with specific markers of HSCs (α-SMA), hepatocytes (CK19), and macrophages (F4/80). The results showed that CD39 was expressed in all three cell types, but the difference was most pronounced in HSC (Supplementary Fig. 1H, I and J). CD73 was most highly expressed in HSCs, and double immunofluorescence staining showed that the CD73-positive area and the α-SMA-positive area in the EtOH-fed+CCl_4_ group highly overlapped (Supplementary Fig. 1K). The expression of CD73 was lower in hepatocytes and macrophages, and the difference between the pair-fed and EtOH-fed+CCl_4_ groups was not evident (Supplementary Fig. 1L and M). Therefore, we selected HSCs as our next study subjects. Furthermore, adenosine levels were higher in the EtOH-fed+CCl_4_ group than in the pair-fed group (Supplementary Fig. 1F). The protein levels of COL1a1, α-SMA, CD39 and CD73were dramatically elevated in primary HSCs isolated from fibrotic livers relative to those in the pair-fed group (Supplementary Fig. 1G). Taken together, these data indicate that CD39 and CD73 are expressed in HSCs and are elevated in the EtOH+CCl_4_-induced mouse model.

Western blotting and immunofluorescence were used to determine the expression of CD39 and CD73 in acetaldehyde-treated HSC-T6 cells. As shown in Supplementary Fig. 2A, the expression of α-SMA and COL1a1 was highest when the acetaldehyde concentration was 200 μM. As shown in Supplementary Fig. 2B, α-SMA and COL1a1 exhibited their highest protein expression levels when the acetaldehyde stimulation time was 48 h. In conclusion, we chose acetaldehyde (200 μM) and 48 h for further studies. Western blot results showed that the expression of α-SMA and COL1a1 in HSC-T6 cells increased after stimulation with acetaldehyde, and the expression of CD39 and CD73 was also significantly increased. Furthermore, the immunofluorescence results of α-SMA, CD39, and CD73 were consistent with those of the western blot analysis (Supplementary Fig. 2C, D, E and F).

### Dynamic changes of CD39-CD73-adenosine axis in the ALF process

To explore the dynamic changes in the CD39-CD73-adenosine axis in the process of ALF, we detected the expression of CD39 and CD73 at 1, 2, 4, 6, and 8 weeks. H&E staining showed that from the second week, the EtOH-fed+CCl_4_ group had increased fat vacuoles and disordered hepatic cords, and the damage was aggravated with time (Supplementary Fig. 3A). Masson staining and Sirius Red staining results showed that mild fibrosis appeared in the liver tissue at 6 weeks, and fibrosis was clear at 8 weeks (Supplementary Fig. 3B and C). This phenomenon was also demonstrated by the expression of COL1a1 and α-SMA in the primary stellate cells and liver tissues (Supplementary Fig. 3D, E, and F). Concurrently, we observed that the expression of CD39 and CD73 increased in the EtOH-fed+CCl_4_ group compared with the pair-fed group from the second week and increased in a time-dependent manner (Supplementary Fig. 3D, E, and F).

### Silencing CD39 and CD73 separately or together attenuated fibrosis induced by acetaldehyde in HSC-T6 cells

Our previous studies have shown that silencing of CD39 or CD73 can alleviate ALF 18, 19. In this study, we aimed to further confirm whether CD39 or CD73 plays the dominant role in ALF. First, the transfection efficiency of CD39-siRNA/CD73-siRNA and pEX3-CD39/pEX3-CD73 was examined by determining CD39 and CD73 protein expression in HSC-T6 cells. As shown in Supplementary Fig. 4A, B, C, and D, when CD39-siRNA/CD73-siRNA was transfected into acetaldehyde- stimulated HSC-T6 cells, the protein expression of CD39/CD73 was significantly decreased. In contrast, when pEX3-CD39/pEX3-CD73 was transfected into acetaldehyde-stimulated HSC-T6 cells, the expression of CD39/CD73 was significantly increased. Taken together, these data indicated that transfection of CD39-siRNA/CD73-siRNA and pEX3-CD39/pEX3-CD73 was successful in HSC-T6 cells. We then silenced CD39 and CD73 separately or together to compare the alleviating effects of CD39 and CD73 on ALF by observing the expression of COL1a1 and α-SMA. The results of protein and mRNA analyses showed that silencing CD39 or CD73 alone could downregulate the elevation of COL1a1 and α-SMA induced by acetaldehyde, and silencing CD73 could significantly reduce the expression of COL1a1 and α-SMA. Furthermore, we found that co-silencing of CD39 and CD73 significantly reduced the expression of COL1a1 and α-SMA, especially when compared to the CD39-siRNA group (Supplementary Fig. 4E and G). In contrast, the overexpression of CD39 and/or CD73 promoted acetaldehyde-induced fibrosis. Interestingly, the expression of COL1a1 and α-SMA was not significantly increased in the pEX3-CD39+pEX3-CD73 group compared to that in the pEX3-CD39 group (Supplementary Fig. 4F and H). These findings indicate that silencing CD73 alone had a better effect on inhibiting the increased expression of COL1a1 and α-SMA induced by acetaldehyde.

### Silencing CD39 and CD73 separately or together facilitated apoptosis of HSC-T6 cells

Subsequently, we compared the effects of CD39 and CD73 on HSC apoptosis. Flow cytometry analyses showed that silencing CD39 or CD73 alone could increase the percentage of apoptotic HSC-T6 cells; the effect of silencing CD73 was more evident, and silencing CD39 further enhanced the apoptosis-promoting effect of CD73 (Supplementary Fig. 5A). The protein expression level of cleaved caspase-3 and the ratio of Bax/Bcl-2 were consistent with the results of apoptosis analysis (Supplementary Fig. 5B). In addition, the expression of cell cycle-related proteins (c-Myc and cyclinD1) also confirmed that silencing CD73 or CD39 alone induced cell cycle arrest in acetaldehyde-stimulated HSC-T6 cells. However, c-Myc and cyclinD1 were not significantly different in the CD73-siRNA+CD39-siRNA group compared to the CD39-siRNA group (Supplementary Fig. 5C). These results suggested that CD73 has a more pronounced effect on HSC apoptosis and proliferation. In contrast, flow cytometry and western blotting results showed that overexpression of CD39 or CD73 can inhibit HSC apoptosis and promote HSC proliferation. The effect of CD73 overexpression alone was more pronounced. However, compared to the pEX3-NC-CD39 group, the pEX3-NC-CD39+ pEX3-NC-CD73 group had no evident influence on the protein expression of cleaved caspase-3, c-Myc and cyclinD1 and the ratio of Bax/Bcl-2 (Supplementary Fig. 5D, E and F). Taken together, these data indicate that CD73 has a more evident effect on reducing the expression of COL1a1 and α-SMA induced by acetaldehyde, promoting the apoptosis of activated HSC, and inhibiting the proliferation of HSC. In this study, we explored the mechanism of action of CD73 in ALF.

### KO of CD73 protected against EtOH+ CCl_4_-induced liver injury and fibrosis in mice

First, we detected fibrosis in the liver tissue of patients with a history of alcohol consumption using Sirius Red staining (Fig. 1A). As shown in Fig. 1B, the CD73 immunostaining signal was increased in liver tissues from liver fibrosis patients with a history of alcohol consumption compared to the normal liver tissues. To further confirm the function of CD73 *in vivo*, CD73 KO mice were used. All mice were genotyped using PCR (Fig. 1C). The absence of CD73 was verified using immunohistochemistry, western blotting, and RT-qPCR (Fig.1E, I and J). H&E and Sirius Red staining showed that CD73 deficiency attenuated EtOH+ CCl_4_-induced liver injury and fibrosis while decreasing serum ALT and AST concentrations (Fig. 1D, G and H). In addition, western blotting and RT-qPCR revealed that the absence of CD73 reduced the expression of α-SMA and COL1a1 (Fig. 1E and J). These results further support an important role for CD73.

### CD73 regulated the p53 signaling pathway

**Figure 2:**
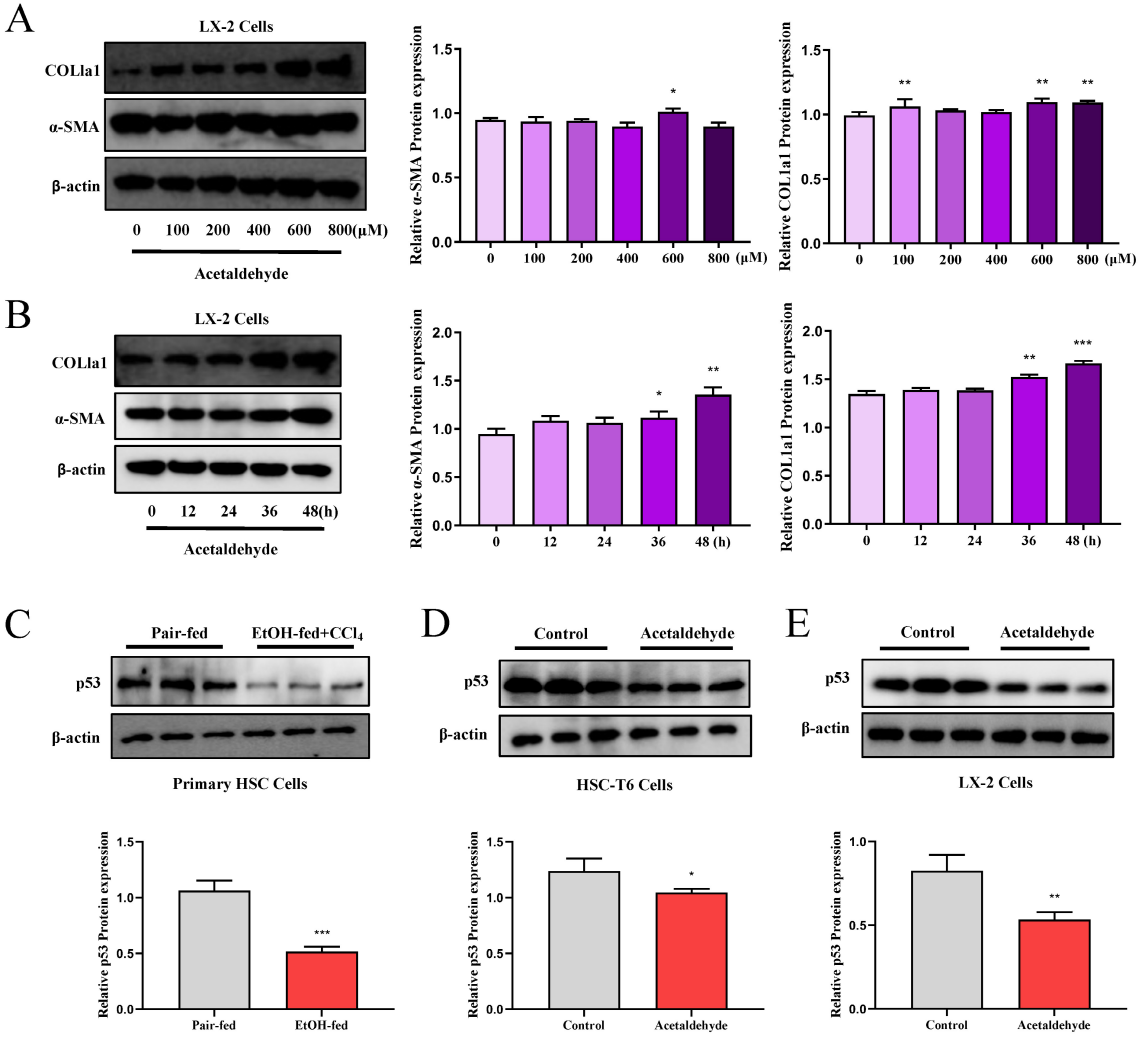
** p53 expression was elevated in EtOH-fed+CCl_4_ mice and in acetaldehyde-stimulated HSC-T6 and LX-2 cells.** Western blot analysis of α-SMA and COL1a1 protein expression at different time periods **(A)** and varying concentrations **(B)**. ^*^P < 0.05, ^**^P < 0.01,^ ***^P < 0.001 vs. the 0 μM/ 0h group. The protein levels of p53 in Primary HSC cells** (C)**, HSC-T6 cells **(D)** and LX-2 cells **(E)**.^ *^P < 0.05, ^***^P < 0.001 vs. the pair-fed/ control group.

**Figure 3:**
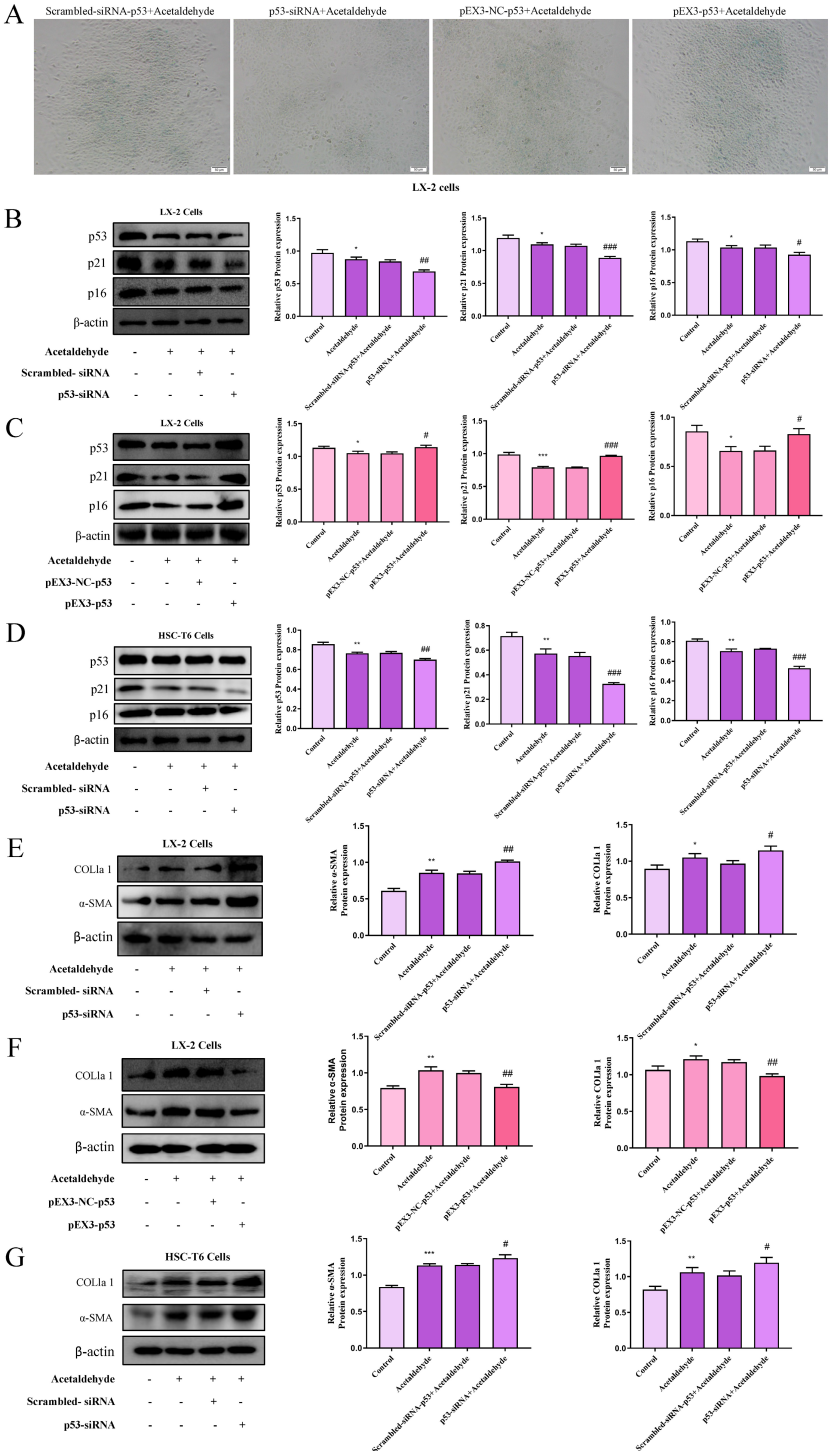
** The p53 signaling pathway regulated activation and senescence of hepatic stellate cells. (A)** p53‐induced senescence as indicated by SA‐β‐Gal activities. Representative images of SA‐β‐Gal staining are presented. **(B, D, E and G)** Western blot analysis of p53, p21, p16, α-SMA and COL1a1 in HSC-T6 and LX-2 cells transfected with p53-siRNA. ^*^P < 0.05, ^**^P < 0.01,^ ***^P < 0.001 vs. the control group. ^#^*P* < 0.05, ^##^*P* < 0.01, ^###^*P* < 0.001 vs. the scrambled- siRNA-p53+Acetaldehyde group. **(C and F)** Western blot analysis of p53, p21, p16, α-SMA and COL1a1 in LX-2 cells transfected with pEX3-p53. ^*^P < 0.05, ^**^P < 0.01,^ ***^P < 0.001 vs. the control group. ^#^*P* < 0.05, ^##^*P* < 0.01, ^###^*P* < 0.001 vs. the pEX3-NC-p53+Acetaldehyde group.

We performed a proteomics assay using HSC-T6 cells (pEX3-NC vs. pEX3-CD73). Kyoto Encyclopedia of Genes and Genomes (KEGG) enrichment analysis revealed that CD73 is associated with the p53 signaling pathway, which is highly associated with cell senescence 20 (Supplementary Fig. 6). First, p53 expression in primary stellate cells was detected by western blotting. The results illustrated that the expression of p53 was significantly reduced in EtOH-fed+CCl_4_ mice compared to that in pair-fed mice (Fig. 2C). Similarly, p53 expression in LX-2 and HSC-T6 cells was tested using western blotting (Fig. 2D and E). We determined acetaldehyde- stimulating conditions for LX-2 cells by examining the expression of α-SMA and COL1a1 at different time periods and concentrations in advance (Fig. 2A and B). To determine the role of the p53 signaling pathway in ALF, we altered p53 expression in acetaldehyde-stimulated LX-2 cells and HSC-T6 cells by transfecting p53 overexpression plasmids or p53 siRNA. Cellular senescence was assessed using senescence-associated beta-galactosidase (SA-β-gal) activity and senescence markers p21 and p16. As shown in Figure 3B, D, E, and G, inhibition of p53 significantly decreased p21 and p16 protein expression and increased α-SMA and COL1a1 protein expression. Furthermore, as shown in Figure 3A, p53 inhibition resulted in a significant decrease in SA-β-gal activity. These results showed that silencing of p53 could inhibit senescence in HSC-T6 and LX-2 cells and promote acetaldehyde- stimulated α-SMA and COL1a1 upregulation. However, overexpression of p53 in LX-2 cells exerted the opposite effect (Fig. 3C and F).

**Figure 4:**
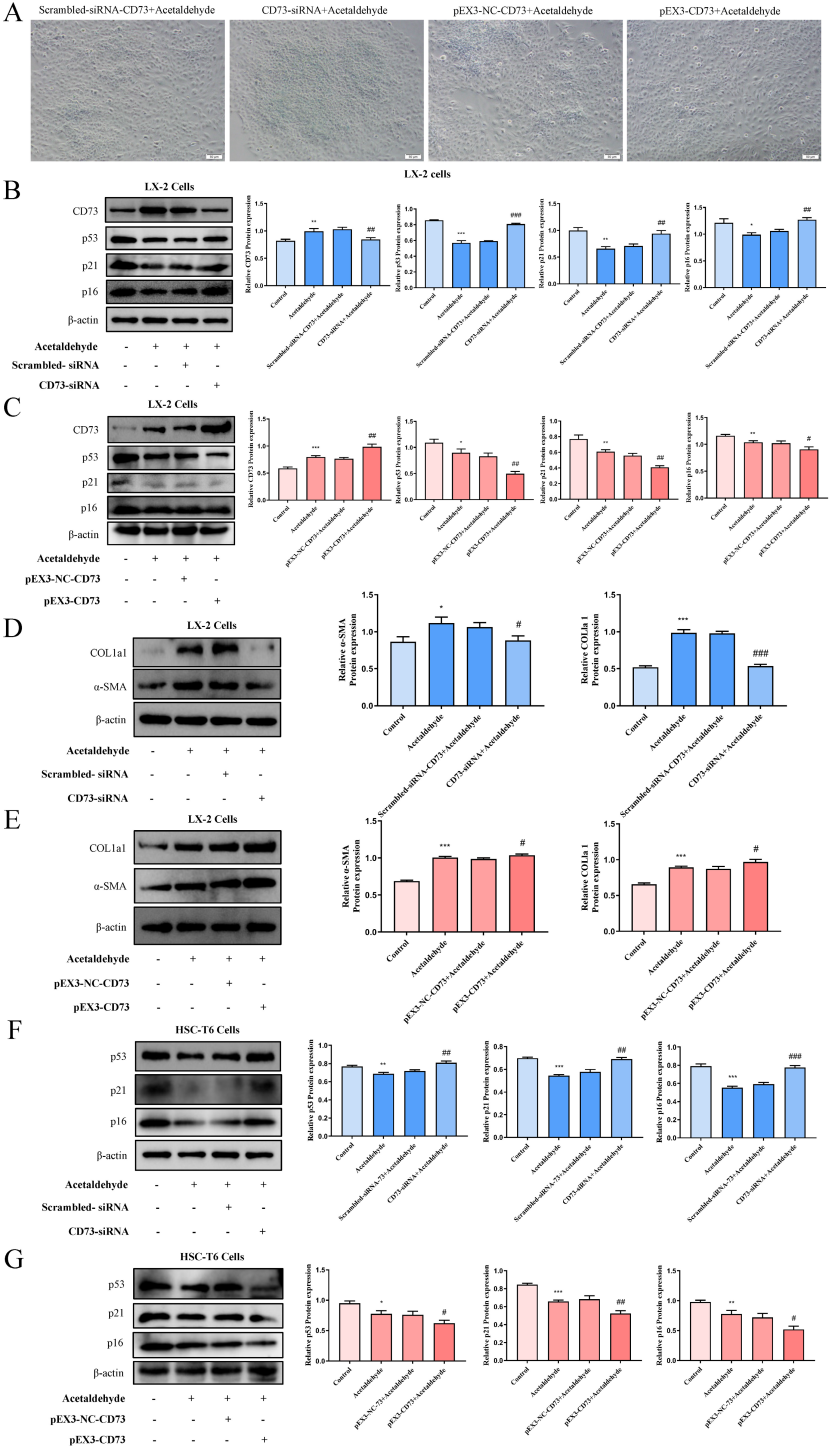
** CD73 regulated the p53 signaling pathway. (A)** Knockdown of CD73 increased the number of SA‐β‐Gal‐positive cells in activated LX‐2 cells. Representative images of SA‐β‐Gal staining are presented. **(B, D and F)** Western blot analysis of p53, p21, p16, α-SMA and COL1a1 in HSC-T6 and LX-2 cells transfected with CD73-siRNA. ^*^P < 0.05, ^**^P < 0.01,^ ***^P < 0.001 vs. the control group. ^#^*P* < 0.05, ^##^*P* < 0.01, ^###^*P* < 0.001 vs. the scrambled- siRNA-CD73+Acetaldehyde group. **(C, E and G)** Western blot analysis of p53, p21, p16, α-SMA and COL1a1 in HSC-T6 and LX-2 cells transfected with pEX3-CD73. ^*^P < 0.05, ^**^P < 0.01,^ ***^P < 0.001 vs. the control group. ^#^*P* < 0.05, ^##^*P* < 0.01 vs. the pEX3-NC-CD73+Acetaldehyde group.

To investigate the regulatory effect of CD73 on the p53 signaling pathway, siRNA-CD73 and pEX3-CD73 were used. Silencing of CD73 increased the protein levels of p53, p21, and p16, while promoting SA-β-gal activity (Fig. 4A, B and F). In addition, the expression of α-SMA and COL1a1 decreased (Fig. 4D and Supplementary Fig. 4E). Conversely, overexpression of CD73 in HSC-T6 and LX-2 cells resulted in a decreased proportion of cellular senescence and increased expression of fibrotic factors (Fig. 4A, C, E, G and Supplementary Fig. 4F). Mechanistically, CD73 regulates hepatic stellate cell senescence via the p53 signaling pathway.

### AURKA was upregulated in liver tissue from patients with a history of alcohol consumption and acetaldehyde-stimulated HSC-T6 and LX-2 cells

**Figure 5:**
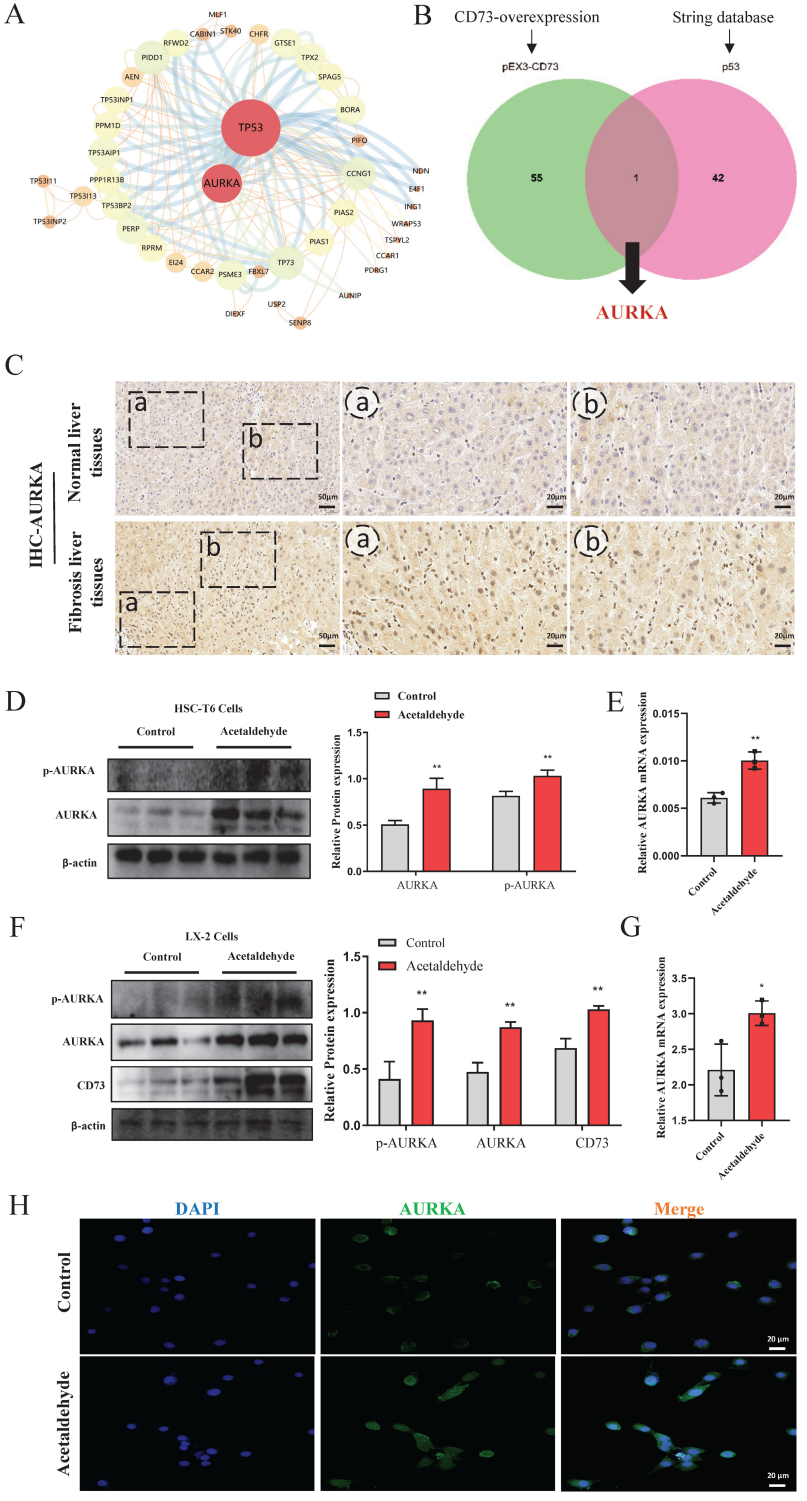
** Expression of AURKA was up-regulated in liver fibrosis patients with a history of alcohol consumption and acetaldehyde-stimulated HSC-T6 and LX-2 cells. (A)** The proteins interacted with p53 were predicted by STRING database. **(B)** The overlap between proteomic and STRING was AURKA. **(C)** Representative pictures of AURKA immune staining were shown (20 μm and 50 μm). **(D)** Western blot analysis of AURKA and p-AURKA in acetaldehyde-stimulated HSC-T6 cells. **(E)** RT-qPCR analysis of AURKA in acetaldehyde-stimulated HSC-T6 cells. **(F)** Western blot analysis of CD73, AURKA and p-AURKA in acetaldehyde-stimulated LX-2 cells. **(G)** RT-qPCR analysis of AURKA in acetaldehyde-stimulated LX-2 cells. **(H)** Immunofluorescent staining of AURKA in acetaldehyde-stimulated LX-2 cells (20 μm).^ *^P < 0.05, ^**^P < 0.01 vs. the control group.

To further investigate the mechanism of CD73 in regulating hepatic stellate cell senescence, we compared the proteomic results of CD73 overexpression with the predicted p53 interacting proteins using the STRING database (Fig. 5A). Proteomic sequencing showed that 56 proteins were regulated after CD73 overexpression. The overlap between the proteomic and STRING databases was Aurora kinase A (AURKA) (Fig. 5B). AURKA is a cell cycle-regulated kinase that plays an important role in hepatocellular carcinoma; however, the role of CD73 in ALF is not known 21. To explore the role of AURKA in ALF, we detected the protein expression of AURKA in patients with liver fibrosis and a history of alcohol consumption, and in HSC-T6 and LX-2 cells stimulated by acetaldehyde. We observed significant changes in AURKA expression in patients with liver fibrosis and a history of alcohol consumption (Fig. 5C). Similarly, the expression of AURKA in acetaldehyde- stimulated HSC-T6 and LX-2 cells showed a tendency to increase compared with the control group (Fig. 5D, E, F and G). We found that the expression of phosphorylated AURKA increased significantly (Fig. 5D and F). In addition, immunofluorescence results revealed that AURKA expression was increased following acetaldehyde stimulation and the nuclear expression levels of AURKA were increased (Fig. 5H).

### AURKA inhibition attenuated high levels of α-SMA and COL1a1 induced by acetaldehyde and promoted cellular senescence

**Figure 6:**
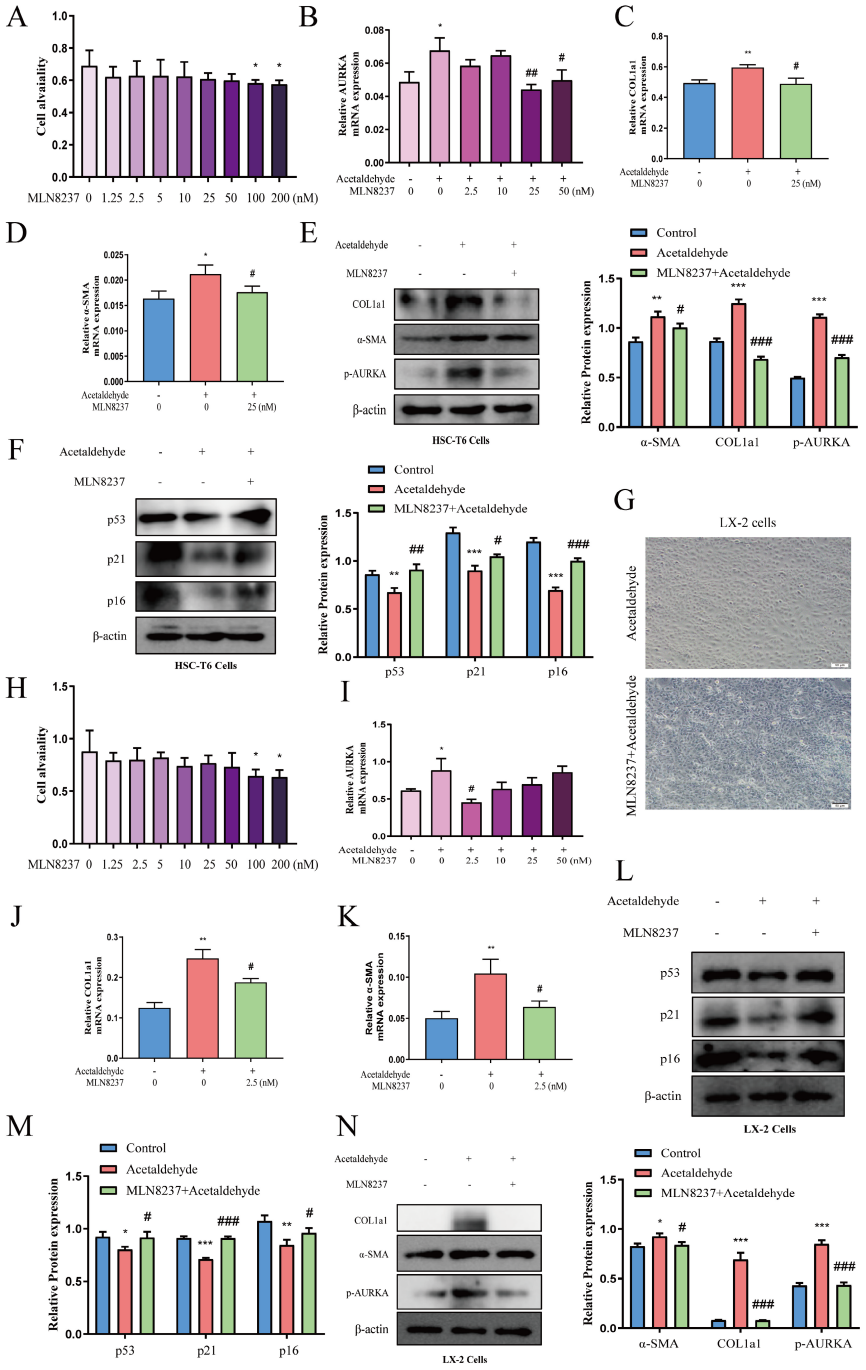
** AURKA inhibition attenuated high levels of α-SMA and COL1a1 induced by acetaldehyde and promoted cellular senescence. (A and H)** Effect of different concentrations of MLN8237 on HSC-T6 and LX-2 cells viability by CCK-8 assay.^ *^P < 0.05 vs. the 0 nM group. **(B and I)** RT-qPCR analysis of AURKA in acetaldehyde-stimulated HSC-T6 and LX-2 cells treated with MLN8237.^ *^P < 0.05 vs. the 0 nM group. ^#^*P* < 0.05, ^##^*P* < 0.01 vs. the acetaldehyde group. RT-qPCR analysis of α-SMA **(D and K)** and COL1a1 **(C and J)** in acetaldehyde-stimulated HSC-T6 and LX-2 cells treated with MLN8237. **(E, F, L, M and N)** Western blot analysis of p53, p21, p16, α-SMA and COL1a1 in acetaldehyde-stimulated HSC-T6 and LX-2 cells treated with MLN8237. ^*^P < 0.05, ^**^P < 0.01,^ ***^P < 0.001 vs. the control group. ^#^*P* < 0.05, ^##^*P* < 0.01, ^###^*P* < 0.001 vs. the acetaldehyde group. **(G)** Inhibition of AURKA increased the number of SA‐β‐Gal‐positive cells in activated LX‐2 cells. Representative images of SA‐β‐Gal staining are presented.

MLN8237, a selective AURKA inhibitor, was added to HSC-T6 and LX-2 cells to further explore the effect of AURKA on HSC activation and senescence. We first evaluated the effect of exposure to different concentrations of MLN8237 (1.25, 2.5, 5, 10, 25, 50, 100, and 200 nM) on cell viability using a CCK-8 assay. The results showed that MLN8237 concentrations higher than 100 nM significantly reduced the viability of HSC-T6 cells (Fig. 6A). Furthermore, the results of RT-qPCR showed that 25 nM MLN8237 was the concentration that had the best inhibitory effect on AURKA (Fig. 6B). In LX-2 cells, we used the same method to select 2.5 nM MLN8237 as the concentration used in the following experiments (Fig. 6H and I). The results of RT-qPCR and western blot analysis revealed that the inhibition of AURKA decreased the expression of p-AURKA, α-SMA, and COL1a1, and significantly increased the expression of p53, p21, and p16 compared with the acetaldehyde group (Fig. 6C-F, J-N). SA-β-Gal staining also showed that cellular senescence increased after MLN8237 treatment in LX-2 cells (Fig. 6G). These results suggest that AURKA inhibition attenuates acetaldehyde-stimulated fibrosis by promoting hepatic stellate cell senescence.

### CD73 regulated the activation and senescence of HSC-T6 and LX-2 cells via modulating ubiquitination of AURKA

**Figure 7:**
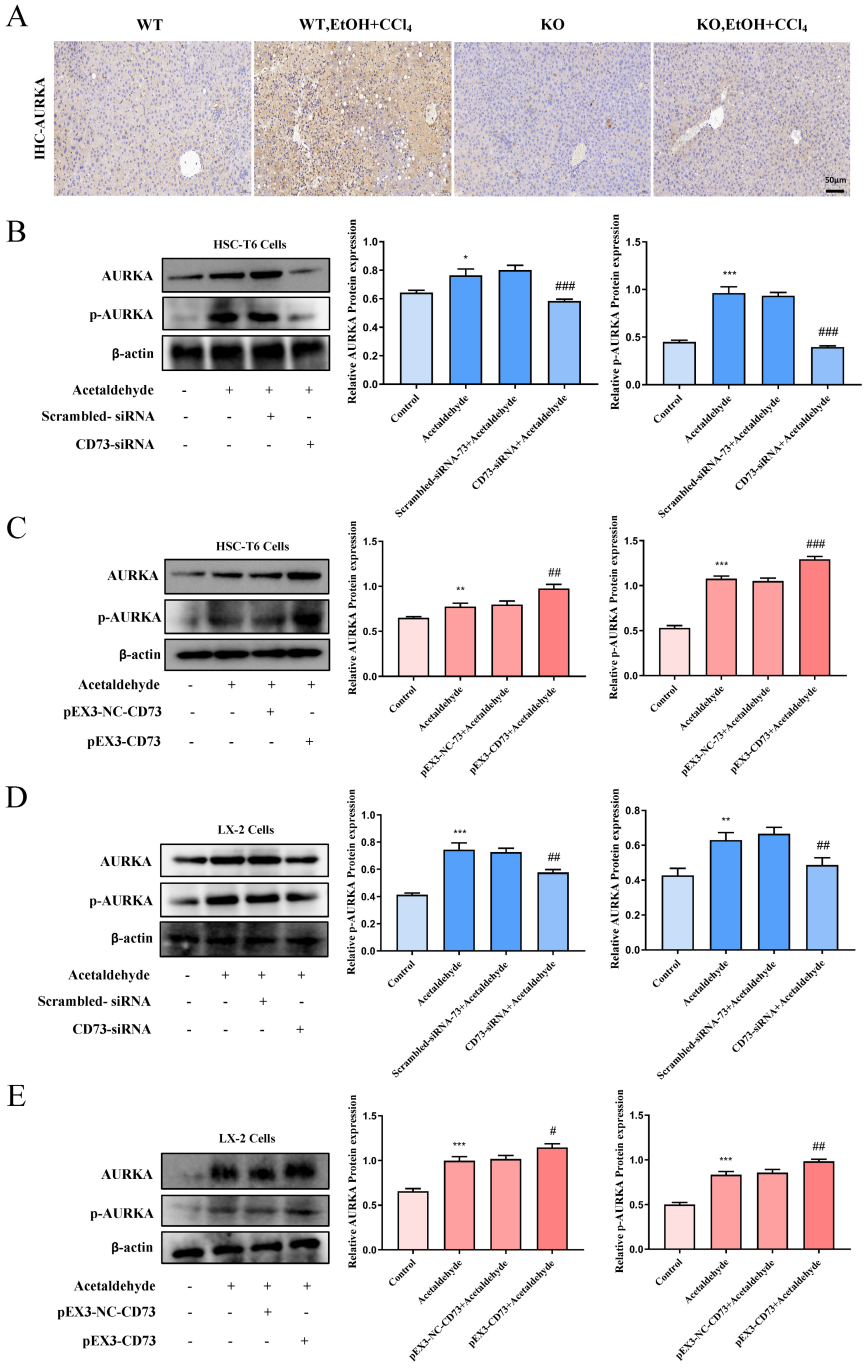
** CD73 regulated AURKA positively. (A)** Representative pictures of AURKA immune staining were shown (50 μm). **(B and D)** Western blot analysis of AURKA and p-AURKA in HSC-T6 and LX-2 cells transfected with CD73-siRNA.^ *^P < 0.05, ^**^P < 0.01,^ ***^P < 0.001 vs. the control group. ^##^*P* < 0.01, ^###^*P* < 0.001 vs. the scrambled- siRNA-CD73+Acetaldehyde group.** (C and E)** Western blot analysis of AURKA and p-AURKA in HSC-T6 and LX-2 cells transfected with pEX3-CD73. ^**^P < 0.01,^ ***^P < 0.001 vs. the control group.^ #^*P* < 0.05, ^##^*P* < 0.01, ^###^*P* < 0.001 vs. the pEX3-NC-CD73+Acetaldehyde group.

**Figure 8:**
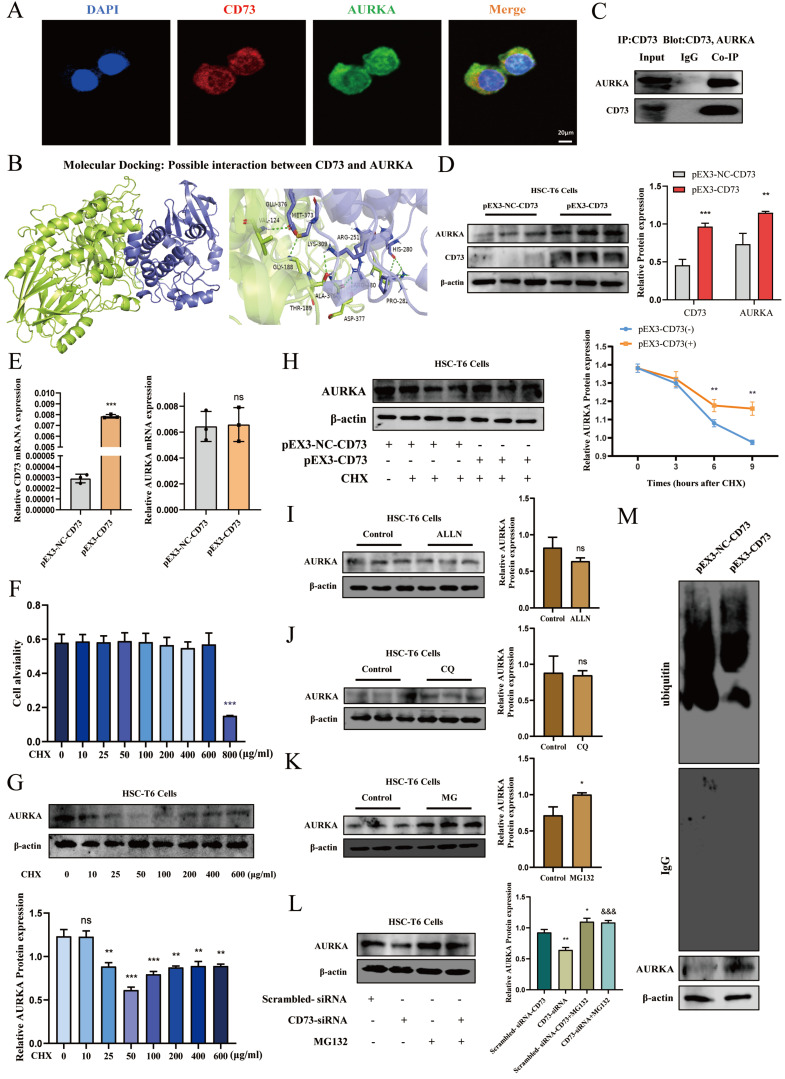
** CD73 increased the stability of AURKA via the inhibition of its ubiquitin/ proteasome-dependent degradation. (A)** Double immunofluorescence staining of CD73 (red) and AURKA (green) (5 μm). **(B)** Molecular docking analysis of molecular interactions between CD73 and AURKA. **(C)** Co-IP of CD73 and AURKA in HSC-T6 cells. The protein **(D)** and mRNA **(E)** levels of CD73 and AURKA in HSC-T6 cells transfected with pEX3-CD73 were measured by Western blot and RT-qPCR. ^**^P < 0.01, ^***^P < 0.001 vs. the pEX3-NC-CD73 group. **(F)** Western blot analysis of AURKA in cells treated with CHX. ^**^P < 0.01, ^***^P < 0.001 vs. the 0 μg/ml group. **(G)** HSC-T6 cells were treated by CHX with or without pEX3-CD73 for 0-9 h. ^**^P < 0.01 vs. the CHX (6 h) or CHX (9 h). Western blot analysis of AURKA in HSC-T6 cells treated with ALLN **(H)**, CQ **(I)** and MG132 **(J)**. ^*^P < 0.05 vs. the control group. **(K)** Western blot analysis of AURKA in HSC-T6 cells treated with MG132 and CD73-siRNA.^ *^P < 0.05, ^**^P < 0.01 vs. the scrambled-siRNA-CD73 group. ^&&&^*P* < 0.001 vs. the CD73-siRNA group. **(L)** Impact of CD73 overexpression on AURKA ubiquitination in HSC-T6 cells.

To determine whether CD73 has a regulatory effect on AURKA, we examined the phosphorylation level and the total expression level of AURKA after knockdown or overexpression of CD73 in HSC-T6 and LX-2 cells and observed that AURKA and p-AURKA protein levels significantly changed with CD73 expression (Fig. 7B, C, D and E). Analysis of immunohistochemical changes in mouse livers confirmed these results (Fig. 7A). Based on the CD73 model, we docked the target protein AURKA using ZDock. According to the ZDock score, ZRank score, and ClusterSize, 13 stable and conventional binding sites between CD73 and AURKA docking were found (Fig. 8B). Immunoprecipitation and double immunofluorescence staining were used to detect interactions between CD73 and AURKA. Immunofluorescence staining showed the co-localization of CD73 and AURKA in HSC-T6 cells (Fig. 8A). Co-immunoprecipitation analysis validated the interaction between CD73 and AURKA (Fig. 8C).

To explore the mechanism underlying the association between CD73 and AURKA, we examined whether the overexpression of CD73 affected AURKA expression and observed that pEX3-CD73 had no significant effect on AURKA mRNA levels (Fig. 8E). However, the overexpression of CD73 increased AURKA protein levels (Fig. 8D). These results suggested that CD73 may affect AURKA protein degradation. First, western blotting was used to determine the concentration of CHX (a protein synthesis inhibitor) in HSC-T6 cells (Fig. 8F). Moreover, following treatment with CHX, CD73 overexpression increased the half-life of the AURKA protein in HSC-T6 cells (Fig. 8G).

The major protein degradation pathways include the ubiquitin-proteasome system (UPS), autophagy-lysosomal pathway (ALP), and Ca^2+^-activated proteases (calpain) 22-24. Chloroquine (CQ, 50 μM), N-[N-(N-Acetyl-L-leucyl)L-leucyl]-L-norleucine (ALLN, 5 μM), and MG-132 (1 μM) were used to inhibit lysosomes, Ca^2+^-activated protease calpain, and proteasomes, respectively. The western blot results indicated that the expression of AURKA was increased in HSC-T6 cells treated with MG132, but not with CQ and ALLN, indicating that the ubiquitin-proteasome pathway mediates the degradation of AURKA (Fig. 8H, I and J). Furthermore, as shown in Fig. 8K, the degradation of AURKA in cells silencing CD73 was blocked by MG132. The results showed that the ubiquitination levels of AURKA were significantly decreased in CD73-overexpressing cells, whereas the ubiquitination levels of AURKA increased in cells that underwent CD73 knockdown (Fig. 8L). Collectively, these results indicated that CD73 increased the stability of AURKA by inhibiting its ubiquitin/proteasome-dependent degradation.

## Discussion

About 3 million people in the Western World die of alcohol-related diseases every year, the most common of which is ALD. Although there are some drugs for the treatment of ALD on the market, the research is still immature, and the treatment is only limited to abstaining from alcohol 25. To date, there are no direct antifibrotic therapies approved for liver fibrosis, and there is a lack of research on the treatment of ALF. One of the main reasons is the difficulty in finding animal models that mimic the human disease process. Simply using alcohol to induce liver fibrosis in mice can only slightly increase the early markers of liver fibrosis, which cannot well represent the formation of ALF 26. Although the use of CCl_4_ alone can induce significant liver fibrosis, it is not consistent with the etiology of human chronic liver disease 27. Therefore, in this experiment, the combination of alcohol and CCl_4_ was used to establish a mouse model to achieve a higher similarity with the human disease 28.

The CD39-CD73-adenosine signaling pathway has emerged as a potential therapeutic target for translational medicine and has received more and more attention, especially in cancer immunotherapy 29-31. Recent studies have demonstrated that CD73 has been identified as a specific immunotherapy target that improves antitumor immune responses to immune checkpoint therapy in glioblastoma 32. CD73 also plays an important role in the progression and metastasis of hepatocellular carcinoma 33. The use of CD39 inhibitors has also been put into clinical trials 34. Adenosine, as an important product of ATP hydrolysis by CD39 and CD73 in the body, participates in the regulation of many physiological and pathological processes in the body by activating adenosine receptors 35. The two sides of purinergic signaling has been reported in Nature 36. Therefore, we observed the expression of CD39 and CD73 during the formation of alcohol-related liver fibrosis. The results showed that the expression of CD39 and CD73 increased in the EtOH-fed+CCl_4_ group from the second week. Interestingly, inhibition of CD39 or CD73 aggravated inflammation 37, 38. Based on the previous experimental results, we believe that the increase of CD39 and CD73 in early-fibrotic-stage was a body's self-protection to liver inflammation. This is consistent with the conclusion confirmed in other studies that although adenosine has a certain anti-inflammatory effect in the short term, the long-term effect can promote the occurrence of fibrosis 39. In future studies we will further explore the specific mechanism of the CD39-CD73-adenosine axis in ALI and ALF. Furthermore, we compared the effects of CD39 and CD73 on the activation and proliferation of HSCs. Western blot, RT-qPCR and flow cytometry results showed that inhibiting CD73 alone can more significantly inhibit the activation and proliferation of HSCs. As a result, we believe that CD73, rather than CD39, was a more critical role in ALF.

The senescence of activated HSCs is an important step in limiting the fibrogenic response to tissue damage. After stopping proliferate, the levels of extracellular matrix-degrading enzymes were increased, while the expression of matrix components was decreased in HSCs 40. The results of proteomic sequencing in HSCs showed that overexpression of CD73 has an impact on the p53 signaling pathway. Multiple lines of evidence indicate that p53 is activated in response to cellular stress, to regulate apoptosis or cell cycle, thereby affecting the aging process 41-43. Studies have shown that the deletion of p53 and the senescence of HSCs contribute to alleviation of CCl_4_-induced liver fibrosis 44, 45. And a recent study demonstrated that adenosine/A_2_B signaling links muscle and brown adipose tissue (BAT) and has both anti-ageing and anti-obesity potential, suggesting that the purine signaling pathway is related to cellular aging 46. In our study, overexpression of p53 induced HSCs senescence with an increase of SA-beta-gal activity, increased expression of p21 and p16, and increased acetaldehyde-induced fibrosis. Furthermore, knockdown of CD73 leads to cellular senescence of HSCs as evidenced by increased SA-beta-gal activity and p21 and p16 protein levels, the protein levels of α-SMA and COL1a1 were also decreased. These results demonstrate that CD73 can regulate fibrosis by regulating cellular senescence.

The regulatory mechanism of CD73 on cellular senescence in ALF was further explored. We found an intersection between the proteomic results of overexpression of CD73 and the results of proteins interacting with p53, that is AURKA. AURKA, a cell cycle-regulated kinase, is an important regulator of mitotic progression and plays an important role in regulating spindle assembly, chromosome segregation, and cytokinesis 47. Activation of AURKA has been shown to have an important role in a broad range of cancers, and its inhibitors have been subjected to clinical trials as monotherapies or in combination with classic chemotherapy or other targeted therapies 48. Recent studies have shown that inhibition of AURKA can reduce renal fibrosis in patients with chronic kidney disease 49. However, the role of AURKA in ALF is still lacking research. Studies have shown that AURKA can participate in the regulation of p53 downstream signaling through multiple pathways, affecting growth arrest and apoptosis pathways 50. MLN8237 can promote cellular senescence and attenuates acetaldehyde-induced fibrosis. As far as we know, this is the first investigation about AURKA in fibrosis progression *in vitro*. We will further investigate the role and mechanism of AURKA in ALF in future studies. Molecular docking data suggested CD73 might bind with AURKA, which was confirmed by Co-IP and immunofluorescence double staining. Meanwhile, we found CD73 stabilized AURKA by inhibiting its ubiquitination and degradation, which is consistent with the reported AURKA degradation pathway 51.

## Conclusions

In conclusion, the current study provides the evidence that overexpression of CD73 inhibits the degradation of AURKA by increasing its interaction with AURKA, thereby inhibiting cellular senescence and aggravating ALF. Therefore, targeting CD39-CD73-adenosine axis may be a promising strategy for the treatment of ALD.

## Supplementary Material

Supplementary figures.

## Figures and Tables

**Table 1:** RT-qPCR primers

Gene	Forward	Reverse
**Rat**		
β-actin	GAGCGCAAGTACTCTGTGTG	CCTGCTTGCTGATCCACATC
CD73	GGCAGATGCTCTTCACAAGG	CCTTCCAGAAGGACCCTGTT
CD39	TTCCTGGTGAGCCTCTCTTG	ATGGCTGAGACGGTTTCTGA
COL1a1	ACCTCAGGGTATTGCTGGAC	GACCAGGGAAGCCTCTTTCT
α-SMA	GAGGGATCCTGACCCTGAAG	CCACGCGAAGCTCGTTATAG
AURKA	CCGAAACGAGTCTTGGTGAC	CTTGAGCACTGGCTTCTGAC
**Mouse**		
GAPDH	AGGTCGGTGTGAACGGATTTG	TGTAGACCATGTAGTTGAGGTCA
CD73	CTGAGCGCTCTACTACCACA	AACAGCACGTTGGGTTCTTC
CD39	GTGTATGTGTGGGTCTGTGC	CCAGCTTGGAAAGACTGACG
COL1a1	TCCCTGGAATGAAGGGACAC	CTCTCCCTTAGGACCAGCAG
α-SMA	ACCCAGCACCATGAAGATCA	TCTGCTGGAAGGTAGACAGC
AURKA	CTGGATGCTGCAAACGGATAG	CGCTGGGAGTTAGAAGGACAC
**Human**		
β-actin	CGCCGCCAGCTCACCATG	CACGATGGAGGGGAAGACGG
CD73	ACAACCTGAGACACACGGAT	TAACTGGGCACTCGACACTT
COL1a1	TCTAGACATGTTCAGCTTTGTGGAC	TCTGTACGCAGGTGATTGGTG
α-SMA	ACGTGGAGCTGTACCAGAAA	GCAGTGTGTTATCCCTGCTG

**Table 2:** siRNA sequences

Gene	Sense (5'→3')	Antisense (5'→3')
**Rat**		
CD73	GGUUGAGUUUGAUGAUAAA	UUUAUCAUCAAACUCAACC
CD39	GGUUGUGAAUGUAAGCGAA	UUCGCUUACAUUCACAACC
p53	GUACUCAAUUUCCCUCAAUTT	AUUGAGGGAAAUUGAGUACTT
Scrambled siRNA	UUCUCCGAACGUGUCACGU	ACGUGACACGUUCGGAGAA
**Human**		
CD73	CAGUUGAAGGUCGGAUCAATT	UUGAUCCGACCUUCAACUGTT
p53	CCACCAUCCACUACAACUATT	UAGUUGUAGUGGAUGGUGGTT
