# Supplementary material for: CD73/NT5E-mediated ubiquitination of AURKA regulates alcohol-related liver fibrosis via modulating hepatic stellate cell senescence
Source: Int J Biol Sci. 2023 Jan 16;19(3):950–66. doi: 10.7150/ijbs.80461 (PMC9909992; doi:10.7150/ijbs.80461)

Supplementary Fig.1 Up-regulation of CD39-CD73-adenosine axis and fibrotic indicators in EtOH+CCl_4 feeding mice. (A) Macroscopic examination of fresh liver tissue without fixation from C57BL/6J mice in pair-fed group and EtOH-fed+CCl_4 group. (B) Serum ALT and AST levels were measured. Representative pictures of H&E (C) and Masson staining (D) of liver tissue sections were shown (50 μm). (E) Representative pictures of α-SMA immune staining were shown (50 μm). (F) Serum adenosine levels were measured. (G) Western blot analysis of CD39, CD73, α-SMA and COL1a1 in primary HSC cells. $^{*}P < 0.05$, $^{**}P < 0.01$, $^{***}P < 0.001$ vs. the pair-fed group. Double immunofluorescence staining of CD39 (red) and α-SMA (H)/ CK19 (I)/ F4/80 (J) (green), representative views from control group and EtOH-fed+CCl_4 mice were presented (50 μm). Double immunofluorescence staining of CD73 (red) and α-SMA (K)/ CK19 (L)/ F4/80 (M) (green), representative views from control group and EtOH-fed+CCl_4 mice were presented (50 μm).

Supplementary Fig.2 Up-regulation of CD39-CD73-adenosine axis and fibrotic indicators in acetaldehyde-induced HSC-T6 cells. Western blot analysis of α-SMA and COL1a1 protein expression at varying concentrations (A) and different time periods (B). $^{*}P < 0.05$, $^{**}P < 0.01$, $^{***}P < 0.001$ vs. the 0 μM/ 0h group. (C) Western blot analysis of CD39, CD73, α-SMA and COL1a1 in acetaldehyde-stimulated HSC-T6 cells. $^{*}P < 0.05$, $^{**}P < 0.01$ vs. the control group. Immunofluorescent staining of α-SMA (D), CD73 (E) and CD39 (F) in acetaldehyde-stimulated LX-2 cells (100 μm).

Supplementary Fig.3 Dynamic changes of CD39-CD73-adenosine axis in the process of ALF. Representative pictures of H&E (A), Masson (B) and Sirius Red staining (C) of liver tissue sections were shown (50 μm). Western blot analysis of CD39, CD73, α-SMA and COL1a1 in primary HSC cells (D) and liver tissues (E). (F) RT-qPCR analysis of CD39, CD73, α-SMA and COL1a1 in liver tissues. $^{*}P < 0.05$, $^{**}P < 0.01$, $^{***}P < 0.001$ vs. the control group.

Supplementary Fig.4 Silencing CD39 and CD73 separately or together attenuated fibrosis induced by acetaldehyde in HSC-T6 cells. Transfection efficiency of CD73-siRNA(A)/ CD39-siRNA (B) and pEX3-CD73 (C)/ pEX3-CD39 (D). The protein (E) and mRNA (G) levels of α-SMA and COL1a1 in HSC-T6 cells transfected with CD73-siRNA/ CD39-siRNA were measured by Western blot and RT-qPCR. The protein (F) and mRNA (H) levels of α-SMA and COL1a1 in HSC-T6 cells transfected with pEX3-CD73/ pEX3-CD39 were measured by Western blot and RT-qPCR. $^{*}P < 0.05$, $^{**}P < 0.01$, $^{***}P < 0.001$ vs. the control group. $^{\$}P < 0.05$, $^{\$\$}P < 0.01$ vs. the scrambled- siRNA-CD39+acetaldehyde/ pEX3-NC-CD39+acetaldehyde group. $^{\#}P < 0.05$, $^{\#\#}P < 0.01$, $^{\#\#\#}P < 0.001$ vs. the scrambled-siRNA-CD73+acetaldehyde/ pEX3-NC-CD73+ acetaldehyde group. $^{\&}P < 0.05$, $^{\&\&\&}P < 0.001$ vs. the CD39-siRNA+acetaldehyde group.

Supplementary Fig.5 Silencing CD39 and CD73 separately or together facilitates apoptosis of HSC-T6 cells. (A and D) The apoptosis of HSC-T6 cells. Western blot analysis of Bax, Bcl-2, Cleaved-caspase3, c-Myc and CyclinD1 in HSC-T6 cells transfected with CD73-siRNA/ CD39-siRNA (B and C) and pEX3-CD73/ pEX3-CD39 (E and F). $^{*}P < 0.05$, $^{**}P < 0.01$, $^{***}P < 0.001$ vs. the control group. $^{\$}P < 0.05$, $^{\$\$}P < 0.01$, $^{\$\$\$}P < 0.001$ vs. the scrambled-siRNA-CD39+acetaldehyde/ pEX3-NC-CD39 +acetaldehyde group. $^{\#}P < 0.05$, $^{\#\#}P < 0.01$, $^{\#\#\#}P < 0.001$ vs. the scrambled-siRNA-CD73+acetaldehyde/ pEX3-NC-CD73+ acetaldehyde group. $^{\&}P < 0.05$, $^{\&\&}P < 0.01$ vs. the CD39-siRNA+ acetaldehyde group.

Supplementary Fig.6 Proteomic analysis of CD73 overexpression in HSC-T6 cells. (A) Volcano plot was presented. GO (B) and KEGG (D) analysis of altered proteins in pEX3-NC-CD73 and pEX3-CD73 overexpressed transfected group. (C) Proteomic was performed with protein extracts from HSC-T6 cells transfected with pEX3-NC-CD73 or pEX3-CD73 overexpressed plasmid.

Supplementary Fig.1

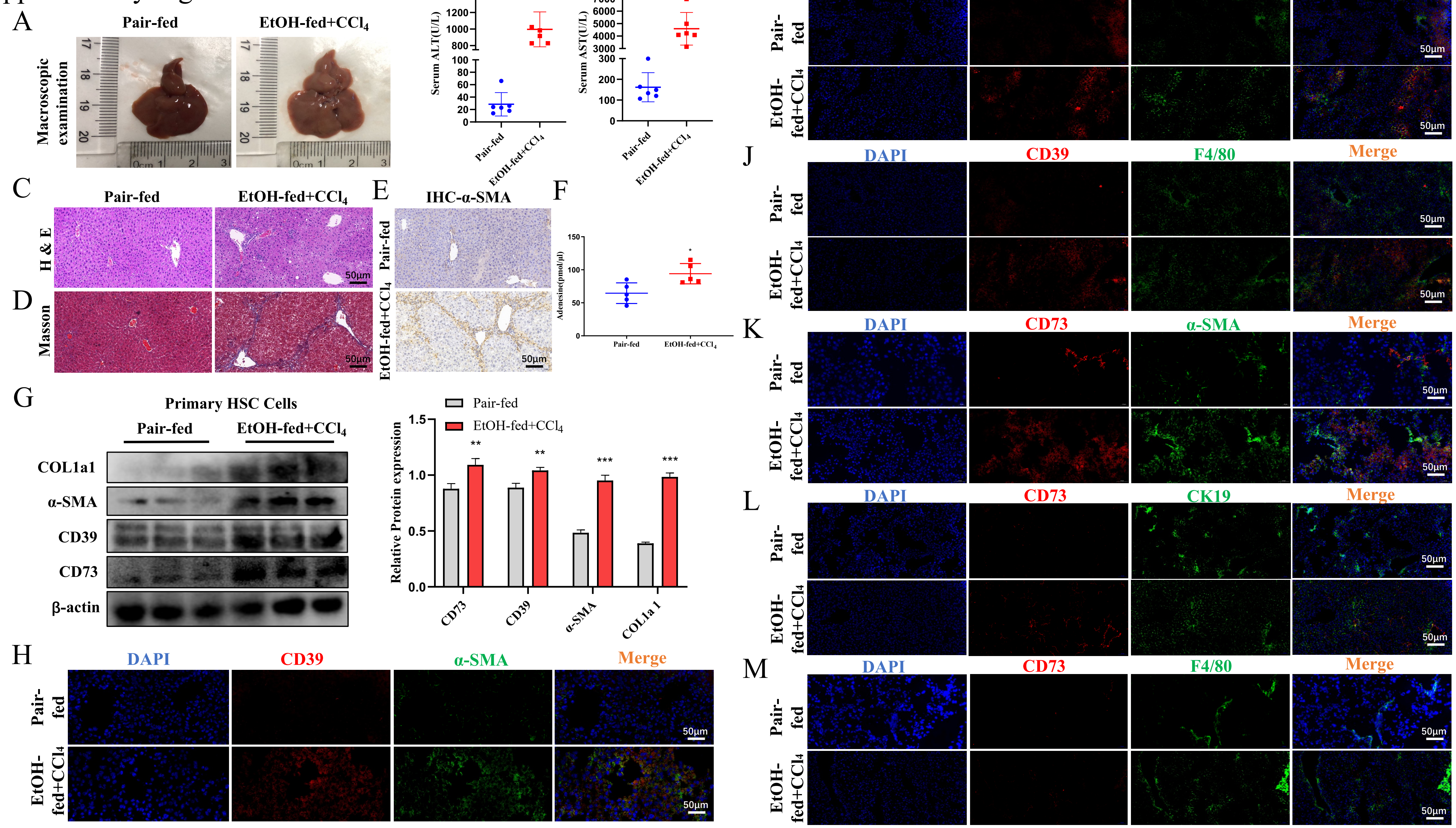

Supplementary Fig.2

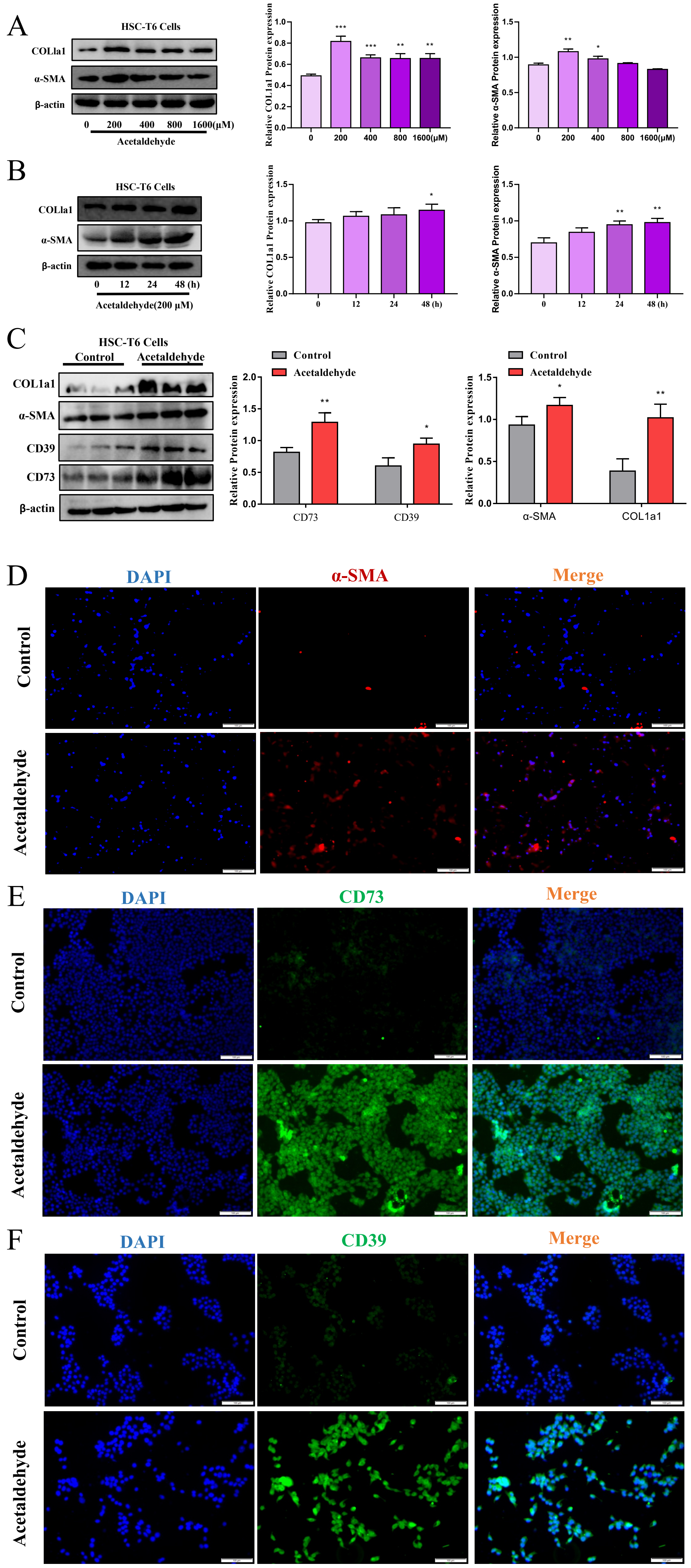

Supplementary Fig.3

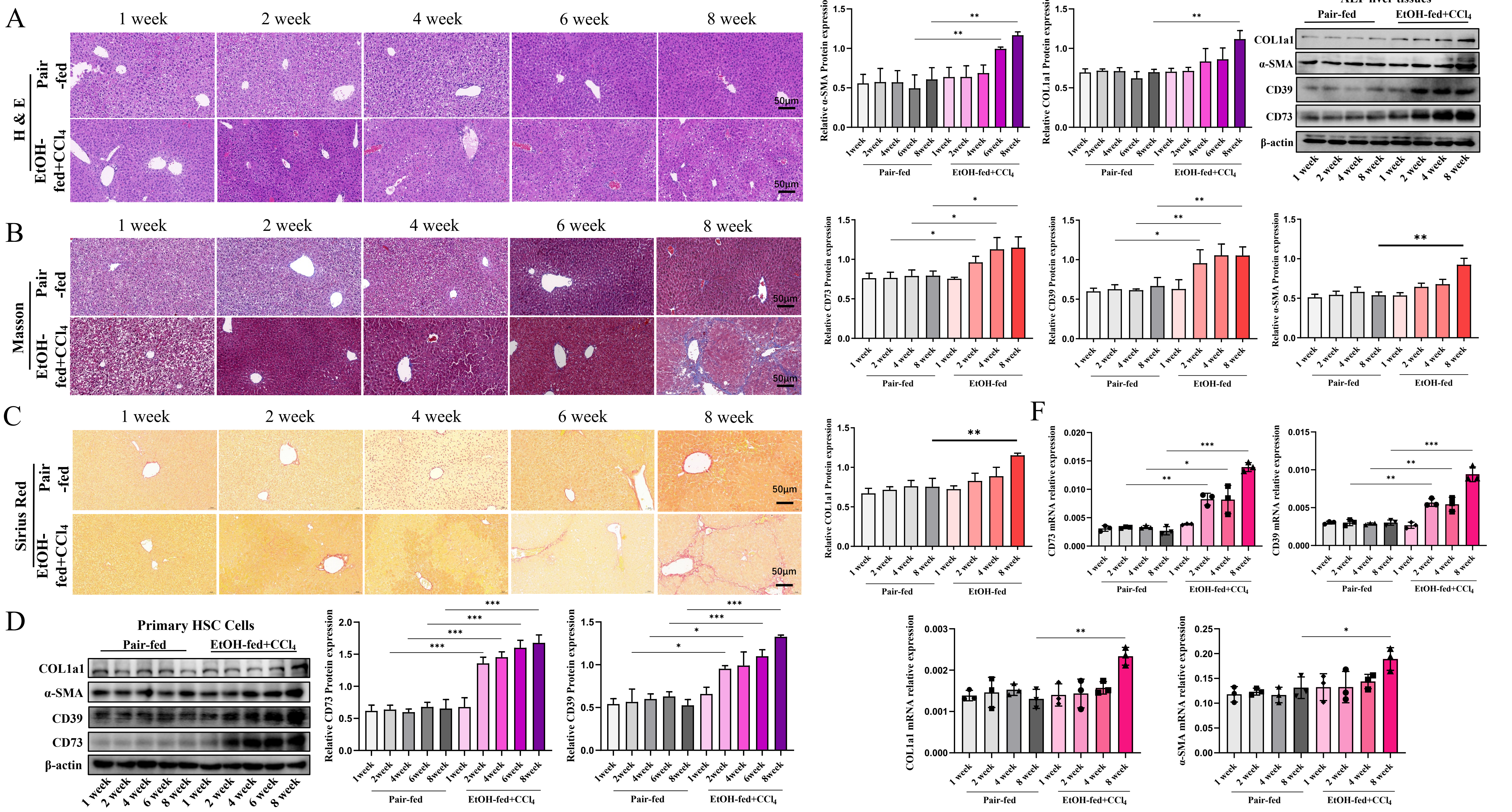

Supplementary Fig.4

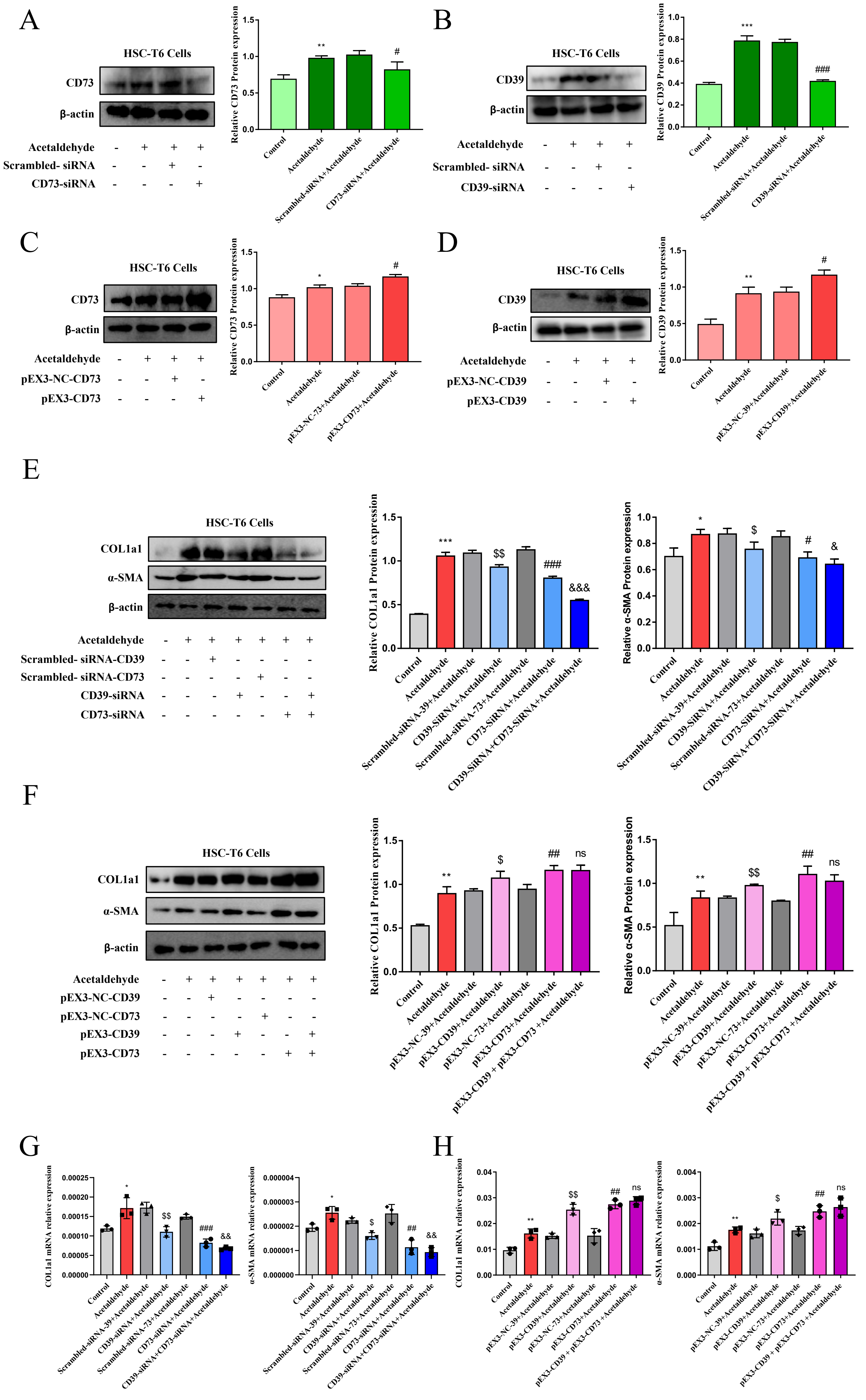

Supplementary Fig.5

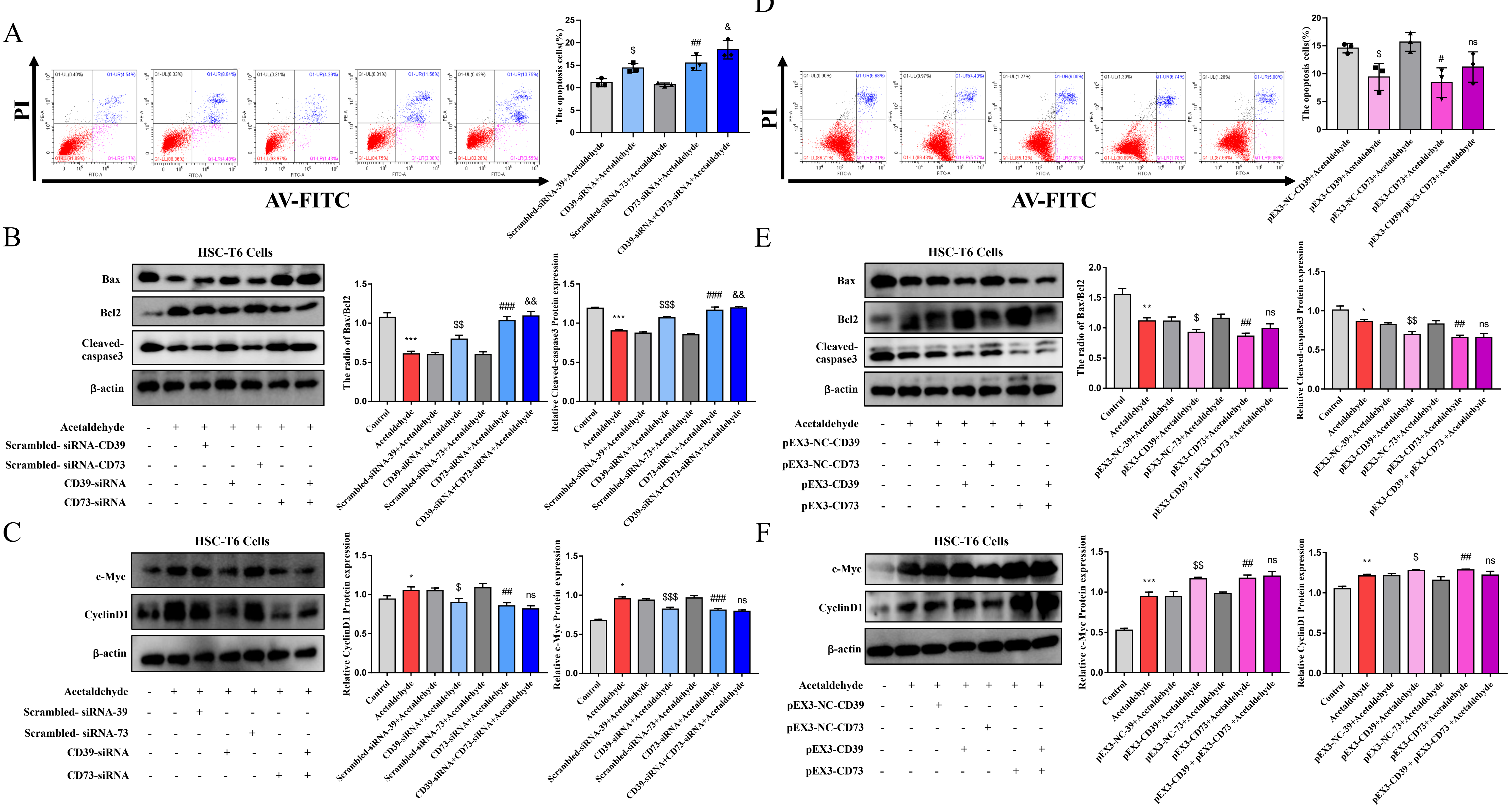

Supplementary Fig.6

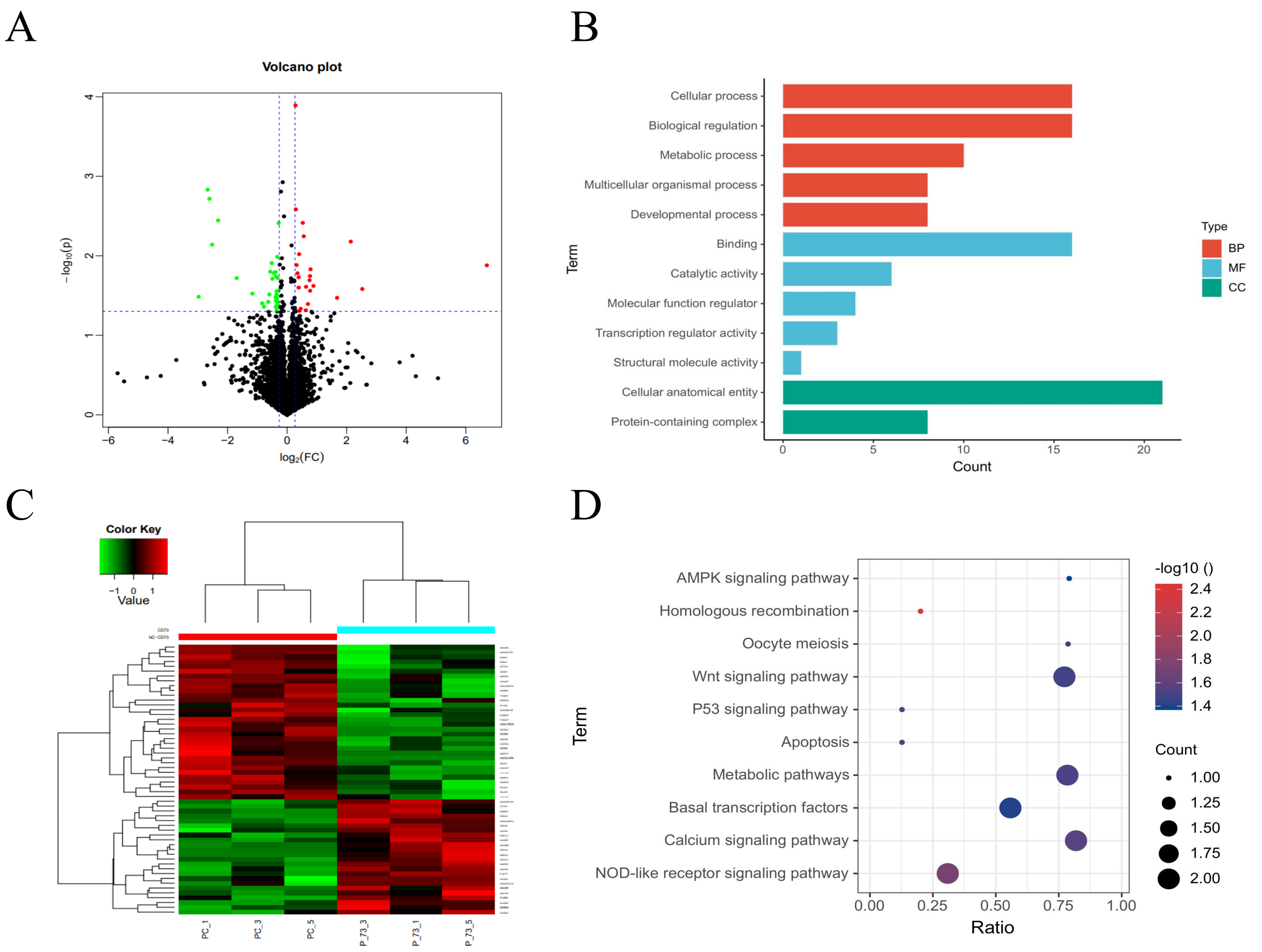

Supplement: Supplementary file 1 — Supplementary figures. [file ijbsv19p0950s1.pdf]
